# Epitope mapping and establishment of a blocking ELISA for mAb targeting the p72 protein of African swine fever virus

**DOI:** 10.1007/s00253-024-13146-x

**Published:** 2024-05-29

**Authors:** Huan-Cheng Liao, Zheng-Wang Shi, Gai-Jing Zhou, Jun-Cong Luo, Wan-Ying Wang, Lu Feng, Fan Zhang, Xin-Tai Shi, Hong Tian, Hai-Xue Zheng

**Affiliations:** State Key Laboratory for Animal Disease Control and Prevention, College of Veterinary Medicine, Lanzhou University, Lanzhou Veterinary Research Institute, Chinese Academy of Agricultural Sciences, Lanzhou, 730000 China

**Keywords:** African swine fever virus, P72, Monoclonal antibodies, Epitope mapping, ELISA

## Abstract

**Abstract:**

The African swine fever virus (ASFV) has the ability to infect pigs and cause a highly contagious acute fever that can result in a mortality rate as high as 100%. Due to the viral epidemic, the pig industry worldwide has suffered significant financial setbacks. The absence of a proven vaccine for ASFV necessitates the development of a sensitive and reliable serological diagnostic method, enabling laboratories to effectively and expeditiously detect ASFV infection. In this study, four strains of monoclonal antibodies (mAbs) against p72, namely, 5A1, 4C4, 8A9, and 5E10, were generated through recombinant expression of p72, the main capsid protein of ASFV, and immunized mice with it. Epitope localization was performed by truncated overlapping polypeptides. The results indicate that 5A1 and 4C4 recognized the amino acid 20–39 aa, 8A9 and 5E10 are recognized at 263–282 aa, which is consistent with the reported 265–280 aa epitopes. Conserved analysis revealed 20–39 aa is a high conservation of the epitopes in the ASFV genotypes. Moreover, a blocking ELISA assay for detection ASFV antibody based on 4C4 monoclonal antibody was developed and assessed. The receiver-operating characteristic (ROC) was performed to identify the best threshold value using 87 negative and 67 positive samples. The established test exhibited an area under the curve (AUC) of 0.9997, with a 95% confidence interval ranging from 99.87 to 100%. Furthermore, the test achieved a diagnostic sensitivity of 100% (with a 95% confidence interval of 95.72 to 100%) and a specificity of 98.51% (with a 95% confidence interval of 92.02 to 99.92%) when the threshold was set at 41.97%. The inter- and intra-batch coefficient of variation were below 10%, demonstrating the exceptional repeatability of the method. This method can detect the positive standard serum at a dilution as high as 1:512. Subsequently, an exceptional blocking ELISA assay was established with high diagnostic sensitivity and specificity, providing a novel tool for detecting ASFV antibodies.

**Key points:**

• *Four strains of ASFV monoclonal antibodies against p72 were prepared and their epitopes were identified.*

• *Blocking ELISA method was established based on monoclonal antibody 4C4 with an identified conservative epitope.*

• *The established blocking ELISA method has a good effect on the detection of ASFV antibody.*

## Introduction

The African swine fever virus (ASFV) is responsible for causing a highly contagious and deadly illness in both domestic pigs and wild boars, known as African swine fever (ASF). ASF is an acute, hemorrhagic, and highly contagious infectious disease, and the fatality rate can reach 100% (Oura et al. 1998). Although the pathogen exhibits a strong affinity for its host and poses no risk of zoonotic transmission, the disease’s recurrent outbreaks and ongoing dissemination have had significant socio-economic ramifications on a global scale (Galindo and Alonso 2017). Since the discovery of ASF in Georgia in 2007, the outbreak in China in 2018 accelerated the spread of ASF and seriously worsened the situation, devastating the pig industry (Zhou et al. 2018; Gaudreault et al. 2020). Currently, there is no effective vaccine against ASF, and intercepting the transmission of ASF mainly relies on culling infected animals (Cadenas-Fernández et al. 2022). Thus, monitoring ASFV antibodies serves as a crucial measure in preventing and managing the disease.

Virus isolation and identification is the classical method for the diagnosing of ASF, but it must be conducted in biosafety level 3 and its time-consuming (Oura et al. 2013). The phenomenon of red blood cell adsorption within 48 to 72 h after inoculation, making the hemadsorption test (HAD) a once-specific detection method for ASFV (Karger et al. 2019; He et al. 2020). However, this method relied on primary pig tissue cell culture, which required 3 to 10 days and had the potential of missing ASFV strains without red blood cell adsorption ability. Currently, PCR-based antigenic testing methods are predominantly used for rapid and specific diagnosis of ASF (de Villiers et al. 2010; Gallardo et al. 2019). Serologic diagnosis of ASF primarily involves ELISA, indirect immunofluorescence test, and immunochromatographic strip technique (Cubillos et al. 2013; Bergeron et al. 2017). Among these methods, ELISA, being the most frequently used antibody detection method, specifically identifies different structural proteins of ASFV including p30, p54, and p72.

The development of effective vaccines and control measures relies on conducting fundamental research on ASFV antigens. The study on the vaccine for ASF explores the subunit vaccine as a highly safe and specific option with great potential (Wu et al. 2020). Nevertheless, the efficacy of immunization using viral structural proteins is frequently inadequate, possibly due to the ability of these proteins to stimulate the production of antibodies that facilitate the virus’s entry into cells, known as antibody-dependent enhancement (ADE) (Gaudreault and Richt 2019). This implies that discovering additional ASFV defensive antigens is a concept aimed at enhancing the immune effectiveness of subunit vaccines. Mass spectrometry has identified a total of 68 structural proteins of African classical swine fever virus (Brookes et al. 1996). P72, which constitutes 31–33% of the entire virion protein, holds utmost significance among them (Wang et al. 2019). The outermost icosahedral capsid is formed by p72 and serves as a highly immunogenic viral protein that can be efficiently utilized as an antigen for diagnostic objectives (Wang et al. 2019; Geng et al. 2022; Caixia et al. 2022). Some studies have shown that tandem p72 epitope fusion protein has potential diagnostic application value (Zhang et al. 2021). Furthermore, the virus attachment to target cells was found to involve the p72 major capsid protein (Duan et al. 2022). The production of antibodies targeting p72 protein and the identification of antigenic epitopes can aid in elucidating the virus's invasion mechanism and potentially serve as a foundation for vaccine development. Up to this point, scientists have discovered certain p72 epitopes (Borca et al. 1994; Heimerman et al. 2018; Yin et al. 2022; Miao et al. 2023). But there are few reports of neutralizing antibodies in these studies, and the application value of the identified epitopes has not been deeply explored.

During this research, we successfully produced and characterized four monoclonal antibodies (mAbs) targeting the recombinant p72 protein produced by *Escherichia coli*. These antibodies show promising potential for practical use. Furthermore, the epitope 20–39 aa exhibit high conservation across all genotypes of ASFV. Subsequently, a blocking ELISA was developed based on the recognition of these conserved epitopes by 72 mAb. The well-established obstructing ELISA exhibits outstanding diagnostic sensitivity and specificity in identifying ASFV antibodies, thus offering a new instrument for detecting ASFV antibody presence.

## Materials and methods

### Cells, plasmids, and reagents

The pET-28a and pMAL-c2x prokaryotic expression vectors were stored in our laboratory. The SP2/0 myeloma cell line and the PAMs, which we maintain in our lab, were cultured in RPMI 1640 medium (Gibco, Thermo Fisher Scientific, Inc) at 37 ℃ in a humidified incubator with 5% CO_2_. All culture media were supplemented with 10% fetal bovine serum (Thermo Fisher Scientific) and antibiotics (0.25 µg/mL amphotericin B, 0.1 mg/mL streptomycin, and 100 IU/mL penicillin; Solarbio Life Sciences, Beijing, China). The maltose-binding protein (MBP) trap column and the Ni Sepharose were purchased from GE Healthcare, USA. The mouse anti-MBP mAbs was purchased from Sigma-Aldrich, Merck, and the ASFV commercial antibody test kit was purchased from Ingenasa, Spain.

### Serum standard and testing samples

The serum samples were utilized in the development and validation of the blocking ELISA. The African Swine Fever Regional Laboratory of China, located at the Lanzhou Veterinary Research Institute, Chinese Academy of Agricultural Sciences, has preserved foot-and-mouth disease (FMDV), porcine reproductive and respiratory syndrome virus (PRRSV), porcine epidemic diarrhea virus (PEDV), pseudorabies virus (PRV), classical swine fever virus (CSFV) infection, and ASFV-positive serum. The ASFV-positive and ASFV-negative sera were verified and preserved by the African Swine Fever Regional Laboratory of China, Lanzhou Veterinary Research Institute, Chinese Academy of Agricultural Sciences (positive and negative sera were distinguished by the method recommended by World Organization for Animal Health).

### Expression and purification of recombinant p72 (rP72)

The ASFV P72 gene sequence was from strain ASFV Georgia 2007/1 (GenBank: FR682468.2), and the prokaryotic expression recombinant plasmid pET-30a-p72 was synthesized and constructed by Wuhan Jinkairui Biology Co, Ltd. The recombinant plasmid pET-30a-p72 was transformed into *E. coli* BL21 (DE3) competent cells (TransGen Biotech, China) and inoculated in Luria–Bertani (LB) liquid medium containing kanamycin (100 μg/mL) and cultured at 37 ℃ and 220 r/min. When the OD_600nm_ value was 0.6–0.8, IPTG (final concentration was 1 mmol/L) was added to induce the expression of 6-h bombardment. Purified rP72 protein was analyzed by SDS-PAGE electrophoresis after purification by Ni Sepharose. The antigenicity of ASFV was identified by western blot method with ASFV inactivated positive serum (1:200) as the first antibody and goat anti-porcine IgG-HRP (1:10,000) as the second antibody.

### Preparation of truncated protein

The truncated p72 gene sequences were amplified from pET-30a-p72 as template and cloned into pMAL-c2x vector for expression. Expression and purification of truncated recombinant plasmids refer to the above, but the transformed *E. coli* inoculated in LB liquid medium containing ampicillin (100 μg/mL) and the expressed proteins were purified using MBP Trap column. The primers used synthesized by Tsingke Biotech (Beijin, China) were listed in Table 1.

**Table 1:** Primers used in this study

Primer names	Primer sequence (5′-3′)	Fragments
A1	F: CGGGATCCTTAGGTACTGTAACGCAGC	1–500 aa
	R: CCAAGCTTACACCCTTAGAGGGCGCCG	
A2	F: CGGGATCCATGCCGATACCACAAGATC	20–303 aa
	R: CCAAGCTTATTCCCTCAGTATCCATTCC	
A3	F: CGGGATCCCTGGGATGCAAAATTTGCGC	430–647 aa
	R: CCAAGCTTATGGCATCAGGAGGAGCTTTTTG	
B1	F: CGGGATCCATGCCGATACCACAAGATC	20–150 aa
	R: CCAAGCTTATGCAGCCCACTCACCACGC	
B2	F: CGGGATCCTGATACCATGAGCAGTTACG	100–220 aa
	R: CCAAGCTTGTAAAACGCGTTCGCTTTTC	
B3	F: CGGGATCCCGGAGATGTTCCAGGTAGG	170–303 aa
	R: CCAAGCTTATTCCCTCAGTATCCATTCC	
C1	F: CGGGATCCATGCCGATACCACAAGATC	20–59 aa
	R: CCAAGCTTCGGGAGGAATACCAACCCAG	
C2	F: CGGGATCCTATAAAATTCTCTTGCTCTG	40–79 aa
	R: CCAAGCTTCGGGGGTTTTAATCGCATT	
C3	F: CGGGATCCGCTTCAAAGCAAAGGTAATC	60–99 aa
	R: CCAAGCTTCTTATCGATAAATTTCCATC	
C4	F: CGGGATCCATGCCGATACCACAAGATC	20–29 aa
	R: CCAAGCTTCCACAAGATCAGCCGTAGT	
C5	F: CGGGATCCGATCAGCCGTAGTGATAGAC	25–34 aa
	R: CCAAGCTTTACGTGGGGTCTATCACTAC	
C6	F: CGGGATCCTAGACCCCACGTAATCCGTG	30–39 aa
	R: CCAAGCTTCGTAATCCGTGTCCCAACT	
D1	F: CGGGATCCAAAGGTTGTGTATTTCAGG	221–226 aa
	R: CCAAGCTTCCCAGTAGACGCAATATACG	
D2	F: CGGGATCCAGATTTCATTAATGACTCC	242–282 aa
	R: CCAAGCTTGATTTGGTGAATGAATTTC	
D3	F: CGGGATCCGTCCAGCTATAAAACGTGAC	263–303 aa
	R: CCAAGCTTATTCCCTCAGTATCCATTCC	

### Preparation of monoclonal antibody against rp72 protein

BALB/c mice aged 6 to 8 weeks were given 100 µg/mouse of the purified rp72 protein. Before vaccination, the protein was mixed with Freund’s complete adjuvant for the first immunization and Freund’s incomplete adjuvant for the following immunizations, which occurred every 2 weeks after the initial one. After euthanizing the mice, spleen cells were gathered 3 days after the last immunization, which was done without emulsification. These spleen cells were then fused with SP2/0 myeloma cells. The cultured fused cells were grown in RPMI 1640 medium with the addition of HAT (Sigma-Aldrich), and then subcloned in the presence of HT (Sigma-Aldrich). The p72-specific antibody produced by the hybridoma was tested using indirect ELISA and confirmed through western blot (WB). To acquire monoclonal hybridoma cell lines and mAbs, the amplified positive clones were subcloned.

### Western blot

In order to examine the reactivity of the anti-p72 monoclonal antibody, rp72 and the truncated protein were isolated using SDS-PAGE and subsequently transferred to an NC membrane using the wet transfer technique. The NC membranes were placed at room temperature (RT) in TBST (0.1% Tween-20) with 5% (w/v) skimmed milk for 2 h. Following that, they were left overnight at 4 ℃ with anti-p72-monoclonal antibody (diluted 1:10,000). The membrane was rinsed with TBST on five occasions, with each rinse lasting at least 5 min. Goat anti-mouse IgG (1:10,000; Abcam, Cambridge, MA, USA) labeled with enzymes was incubated at room temperature for 1 h. Following 5 rounds of washing the film with TBST, the HRP color development was performed using ECL luminescent solution. The following WB steps were identical to those executed for the previous ones.

### Indirect ELISA

To test the anti-p72 hybridoma supernatant, purified rp72 was diluted into a carbonate coating buffer solution (pH9.6) with a concentration of 2 μg/mL and 50 μL/well-coated polystyrene plate. Each well was incubated at 37 ℃ for 30 min after the addition of hybridoma supernatant. After washing four times with PBST, Goat anti-mouse IgG-HRP (1:10,000) was introduced into every well and left to incubate at a temperature of 37 ℃ for a duration of 30 min. The PBST solution underwent four rounds of washing, followed by the addition of tetramethylbenzidine (TMB, 50 μL/well). The mixture was then incubated at a temperature of 37 ℃ inside a dark enclosure for a duration of 12 min. Then, 10% sulfuric acid was used to halt the reaction, and the absorbance was subsequently measured at a wavelength of 450 nm. To locate the epitopes of each monoclonal antibody, purified truncated p72 fragments were coated on a plate with monoclonal antibody (1:2000) as primary antibody and an unrelated monoclonal antibody as negative control. The remaining procedures are identical to those of the hybridoma supernatant.

### Immunofluorescence assay

In order to detect positive hybridoma, an IFA assay was conducted on PAMs that were infected with the ASFV CN/GS/2018 strain (1 MOI). The cells were fixed with precooled 4% paraformaldehyde for 20 min, 48 h after being infected. Subsequently, they were permeated with 0.1% Tryton and sealed using 5% BSA. Rinse using PBS three times after every stage, then expose to hybridoma supernatant at a temperature of 37 ℃ for a duration of 30 min. Following the washing process, add FITC-coupled Goat Anti-Mouse IgG (Abcam) diluted with PBS at a ratio of 1:100 and incubated in the dark. After incubation overnight at 4 ℃, DAPI (1:2000, Beyotime Biotechnology, China) was used to stain the nuclei, which were then imaged using EVOS FL fluorescence microscopy (Thermo Fisher Scientific).

### Procedure for ASFV blocking ELISA

The 4C4 mAb was purified and then coupled with horseradish peroxidase (HRP) obtained from Roche, Inc. Different conditions were examined to investigate the blocking ELISA conditions for the primary stages. Checkerboard titration tests were utilized to optimize the conditions. Following optimization, the purified recombinant p72 protein constructs were applied onto flat-bottom polystyrene plates (50 ng/well) using carbonated coating buffer (pH 9.6). The plates were then incubated at 4 ℃ overnight. The plate was rinsed four times using PBST (0.05% Tween in PBS), then blocked with 5% BSA at 4 ℃ overnight. Following the drying process, 50 μL of both control serum and the serum to be tested were added to each well. The mixture was then incubated at a temperature of 37 ℃ for a duration of 30 min. Following the washing process, 50 μL of 4C4-HRP monoclonal antibody with the appropriate concentration was introduced into every well and left to incubate at a temperature of 37 °C for a duration of 30 min. Following the washing process, 50 μL of TMB solution was introduced into every well and left to incubate at a temperature of 37 ℃ for a duration of 10 min. In the end, 50 μL of termination solution were added to each well in order to stop the reaction. The absorption value was read at 450 nm. The controls consisted of a serum sample with positive results, a serum control with negative results, and a control with buffer. The interpretation of the blocking ELISA results was based on the percent of inhibition (PI) value. The OD value was converted through the following formula: PI (%) = [(1 − OD450 value of sample /OD450 value of negative controls)] × 100%.

### Cut-off value of blocking ELISA

To determine the cut-off value, 154 serum samples were tested by blocking ELISA, comprising 87 serums were negative for ASFV and 67 serums were positive for ASFV. The calculation of the PI value for each detection result, the drawing of the ROC curve, and the calculation of the Youden index for each cut-off value were performed. When the Youden index is the maximum value, it is determined to be the cut-off value of the method.

### Diagnostic sensitivity, specificity, and reproducibility determination

To assess the specificity of this technique, we detected the presence of positive serum of FMDV, PRRSV, PEDV, PRV, CSFV, and ASFV using established blocking ELISA procedures. To assess the method sensitivity, two ASFV-positive sera were serially diluted from 1:4 to 1:1024 using the established ELISA protocol. This method and a commercial ASFV-blocking ELISA kit were used to detect the aforementioned diluted samples simultaneously, and a statistical comparison was made to assess the sensitivity of the two samples. To determine reproducibility, four enzyme-label plates were used to detect three ASFV-positive serum samples and three ASFV-negative serum samples in different batches. Each sample was tested with four replicates. Intra-batch repeats were performed on the same enzyme-label plate, and inter-batch repeats were performed between different enzyme-label plates, so as to calculate the coefficient of variation by blocking rate.

### Evaluation of antibody dynamics

The method was used to track the antibody response of animals immunized with ASFV attenuated live vaccine. Serum from 5 immunized pigs and 1 control pig were collected and separated every 7 days. The antibody levels of all serum samples were detected and analyzed, and the antibody curve was drawn.

### Clinical sample test results

The established blocking ELISA method and the commercial ASFV blocking ELISA kit produced by Ingenasa in Spain were used to detect 150 clinically collected pig serum samples (collected before the outbreak of ASF) and 39 inactivated ASF positive swine serum samples at the same time. The detection results of the two methods were compared, and the coincidence rate of the two methods was calculated.

### Statistical analysis

Statistical analysis was performed using GraphPad Prism. All experiments were repeated thrice.

## Results

### Identification of rp72

**Fig. 1:**
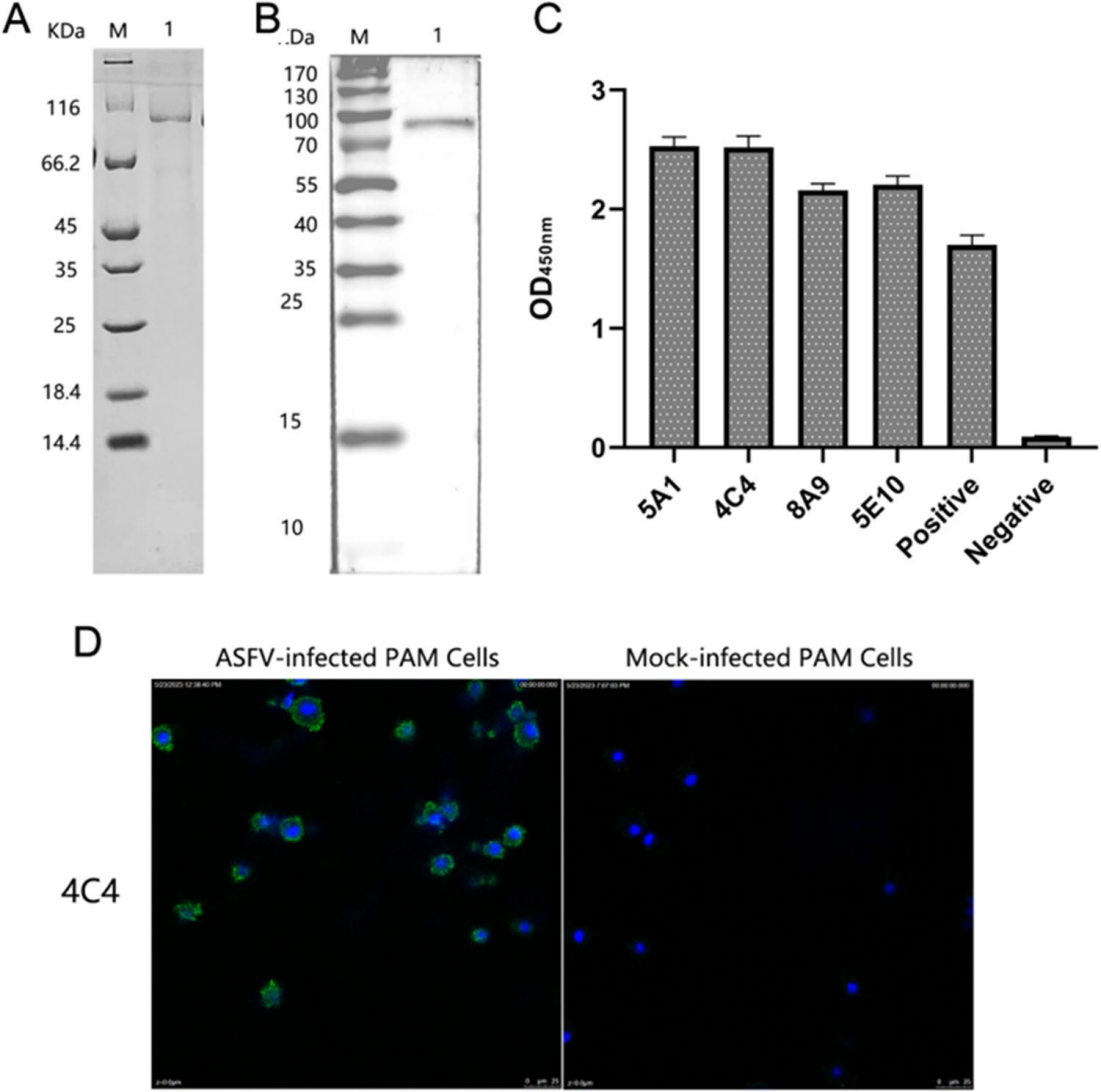
Characterization of rp72 protein and mAbs. **A** An analysis was conducted on the recombinant rp72 protein using SDS-PAGE. The 81 kD recombinant protein is visible. **B** Western blot analysis of recombinant rp72 protein with ASFV positive serum. The 81 kD recombinant protein is visible. **C** Indirect ELISA detection of hybridoma cell supernatant. Purified rp72 was applied to coat the plate, followed by incubation with the hybridoma supernatants as the primary antibody and HRP-conjugated goat anti-mouse IgG as the secondary antibody. The negative control consisted of SP2/0 cell supernatant. **D** The IFA method was used to analyze the reactivity of 4C4 mAbs. At 48 h post-infection, the cells were immobilized. Hybridoma supernatants were used as the primary antibody, while FITC-conjugated goat anti-mouse IgG served as the secondary antibody during cell incubation. The nucleus of PAM cells is stained blue by DAPI

The fusion protein rp72 was expressed in *Escherichia coli* and predominantly existed as a soluble form. Purification of rp72 was achieved using a His-tagged protein purification kit, resulting in highly pure protein with an apparent molecular weight of approximately 81 kDa as confirmed by SDS-PAGE analysis (Fig. 1A). Western blotting further demonstrated the reactivity of purified rp72 with ASFV positive serum (Fig. 1B).

### Characterization of anti-p72 mAbs

By performing limiting dilution and indirect ELISA to test supernatants, four hybridoma cell lines were generated that stably secrete mAbs against the p72 protein after undergoing subcloning three times. ELISA and western blot results showed 4 mAbs respond well to rp72 (Fig. 1C). IFA results showed that 4C4 monoclonal antibody was highly reactive to ASFV-infected cells (Fig. 1D).

### Epitope mapping

**Fig. 2:**
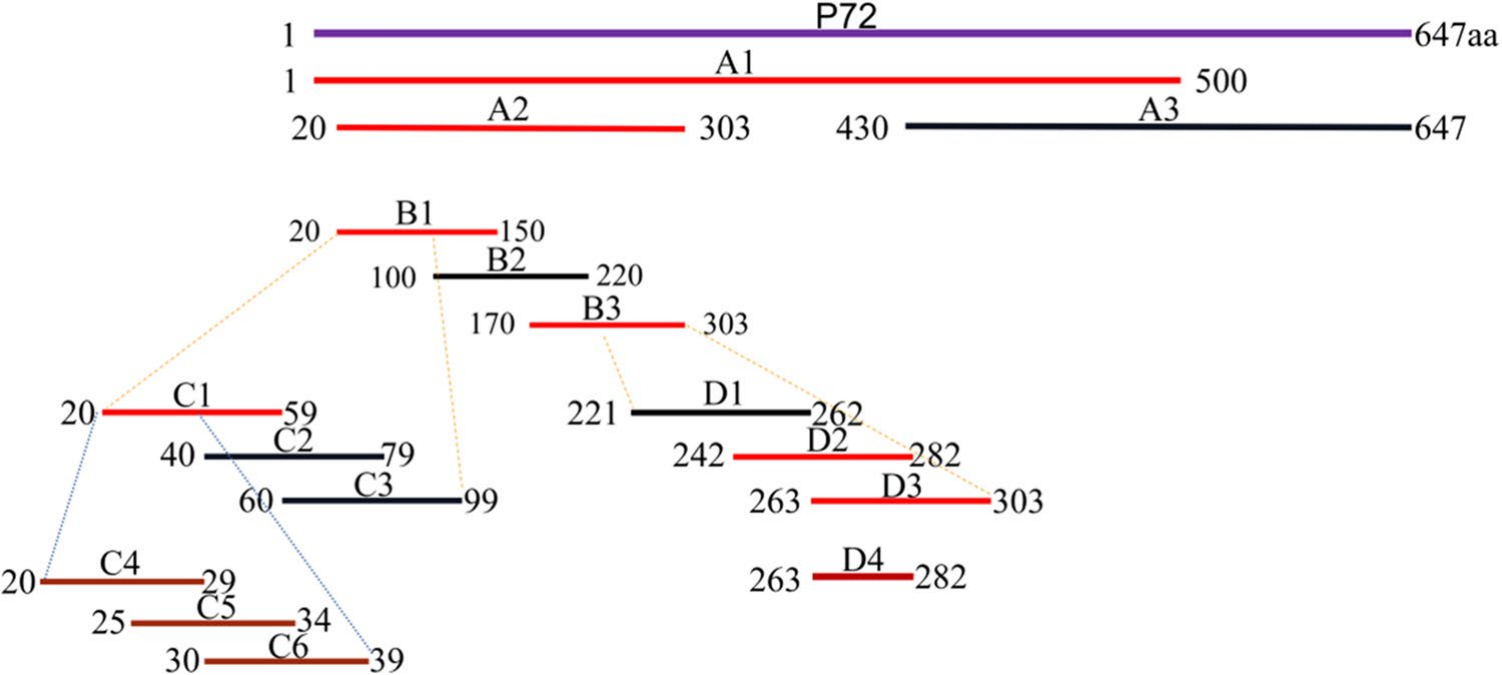
Schematic representation of p72 fragments used for epitope mapping. The truncated fragment diagram of the antibody recognition epitope is gradually located, and the lower peptide is truncated from the upper peptide, in which the truncated peptide recognized by the monoclonal antibody is marked red

**Fig. 3:**
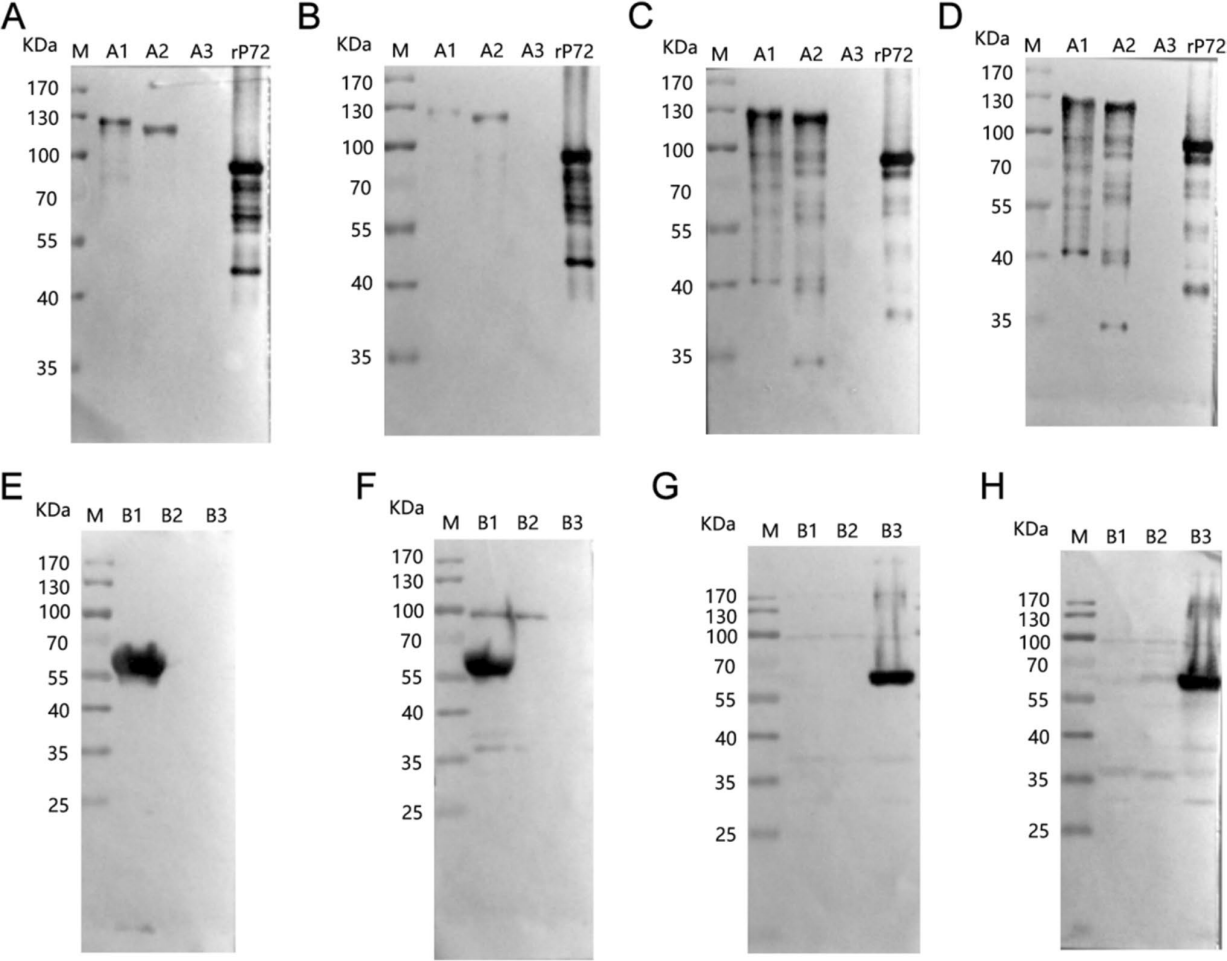
Preliminary mapping of mAb epitopes. **A**–**D** Preliminary mapping of the 4C4, 5A1, 5E10, and 8A9 epitope with western blot. In the initial mapping, the complete rp72 protein was shortened to peptides A1 (1–500 aa), A2 (20–303 aa), and A3 (430–647 aa). A1, A2, and A3 were produced as MBP-fused proteins in *E. coli*. **E**–**H** To further map the 4C4, 5A1, 5E10, and 8A9 epitopes, western blotting was conducted. A2 was truncated into peptides B1 (20–150 aa), B2 (100–220 aa), and B3 (170–303 aa) for the mapping process. MBP-fused proteins of B1, B2, and B3 were then expressed in *E. coli*. M: protein weight marker for molecular

To locate epitopes for each mAbs, the p72 protein is progressively truncated to A–D, as shown in Fig. 2. To determine the exact epitopes acknowledged by the four monoclonal antibody variants and guarantee the preservation of all recognition sites, we initially partitioned p72 into three sections: A1 (amino acids 1–500), A2 (amino acids 20–303), and A3 (amino acids 430–670). Western blot results demonstrated that antibodies 8A9, 5A1, 4C4, and 5E10 all recognized both A1 and A2 but showed no reactivity towards A3 (Fig. 3A–D). This suggests that these antibodies are localized within amino acids 20–303. Subsequently, we further subdivided amino acids 20–303 into B1 (amino acids 20–150), B2 (amino acids 100–220), and B3 (amino acids 170–303). The Western blot analysis revealed that only 5A1 and 4C4 reacted with B1, while 8A9 and 5E10 specifically bound to B3 (Fig. 3E–H). Specifically, 5A1 and 4C4 targeted amino acid positions within the range of 20–99, whereas 8A9 and 5E10 recognized residues between 221 and 303.

**Fig. 4:**
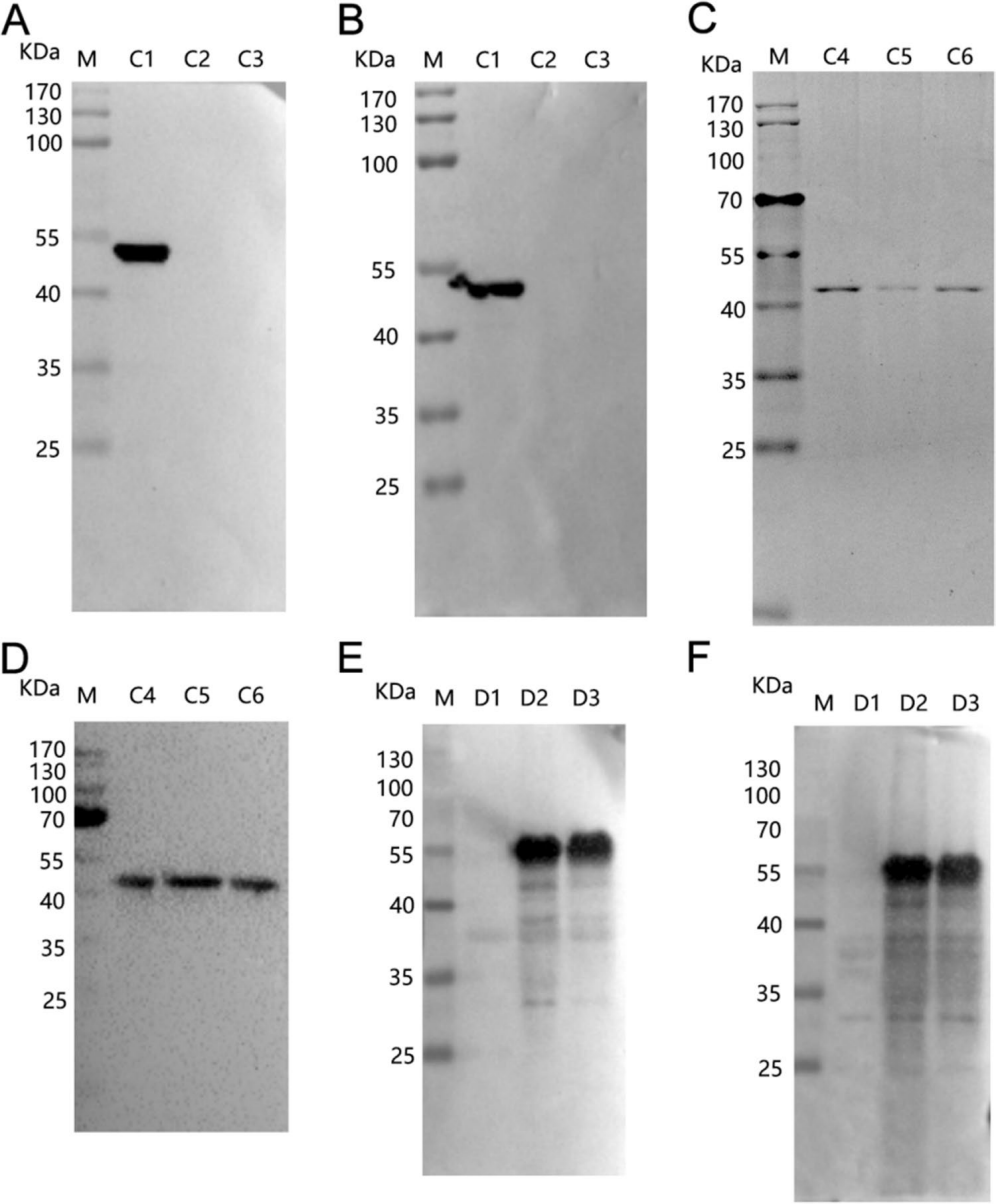
Precise mapping of the mAb epitope. **A**, **B** The initial stage of accurate epitope mapping for 4C4 and 5A1 was conducted using western blotting. We divided 20–99 aa into three overlapping peptides, C1 (20–59 aa), C2 (40–79 aa), and C3 (60–99 aa). **C**, **D** The western blot technique was used for the second round of accurate epitope mapping of 4C4 and 5A1. The unique region (20–39 aa) of C1 was divided into three overlapping peptides, C4 (20–29 aa), C5 (25–34 aa), and C6 (30–39 aa). **E**, **F** The precise epitope identification of 5E10 and 8A9. We divided 221–303 aa into three overlapping peptides, D1 (221–262 aa), D2 (242–282 aa), and D3 (263–303 aa). The peptides C1-C6 and D1-D3 were inserted into the pMAL-c2x plasmid and produced as MBP-fused proteins in *E. coli*. The shortened peptides were identified using the corresponding monoclonal antibody. M: protein weight marker for molecular

In order to identify the precise epitope acknowledged by 5A1 and 4C4, the amino acids ranging from 20 to 99 were segmented into peptides C1, C2, and C3, and only C1 exhibited recognition by both 5A1 and 4C4 (Fig. 4A, B). Further, the unique region of C1, 20–39 aa, was divided into C4, C5, and C6 segments. And all three segments were recognized by 5A1 and 4C4 (Fig. 4C, D). Therefore, it was definitively established that the antigenic determinant identified by 4C4 and 5A1 is situated within the amino acid sequence spanning residues 20 to 39.

In order to accurately determine the specific location of the epitope identified by 8A9 and 5E10, we systematically shortened the distinct portion of B3 (221–303 aa) into peptides D1–D3. The findings indicated that peptides D2 and D3 exhibited a reaction with 8A9 and 5E10 (Fig. 4E, F). It is determined that the epitopes identified by 8A9 and 5E10 are located in the 263–282 aa region that overlaps D2 and D3. This coincides with the reported epitopes of 265–280 aa in p72.

### Homology analysis of p72 epitopes

**Fig. 5:**
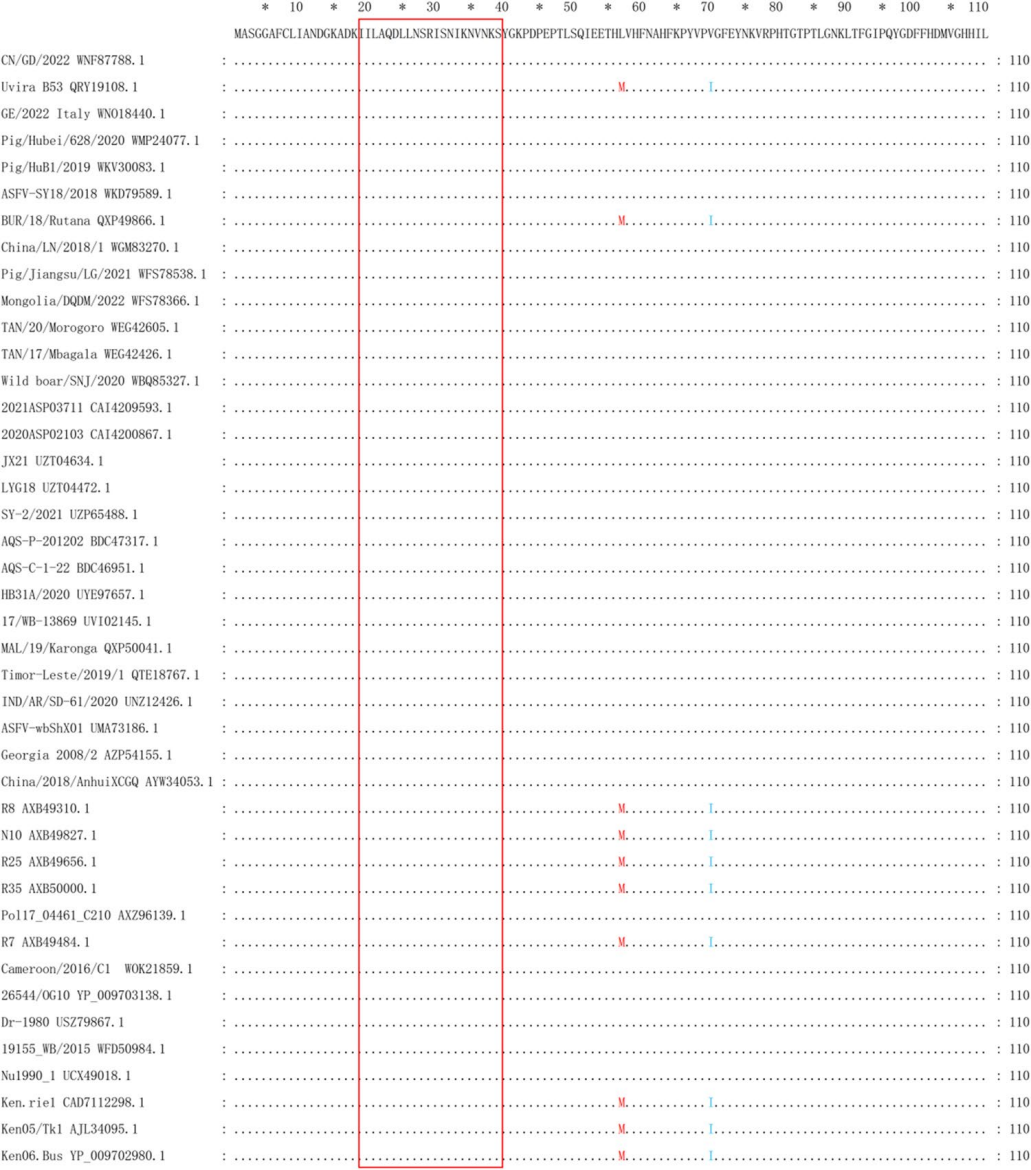
Sequence alignment of p72 proteins of different strains. The p72 sequences of 42 ASFV strains were aligned with GeneDoc. Black dots represent matching residues, while dashed lines indicate gap regions. The alignment specifies the amino acid’s coordinate on the top and right terminus for each sequence. Epitope regionsof 20–39 aa recognized by 4C4 are circled with red

To determine the conservation of the resulting epitope region across different ASFV genotypes, we conducted an analysis on the p72 sequence of 42 ASFV isolates. As depicted in the figure, there is a high degree of amino acid alignment conservation within the sequence where epitopes are located (Fig. 5).

### Standardization and determining the cut-off value for blocking ELISA

**Fig. 6:**
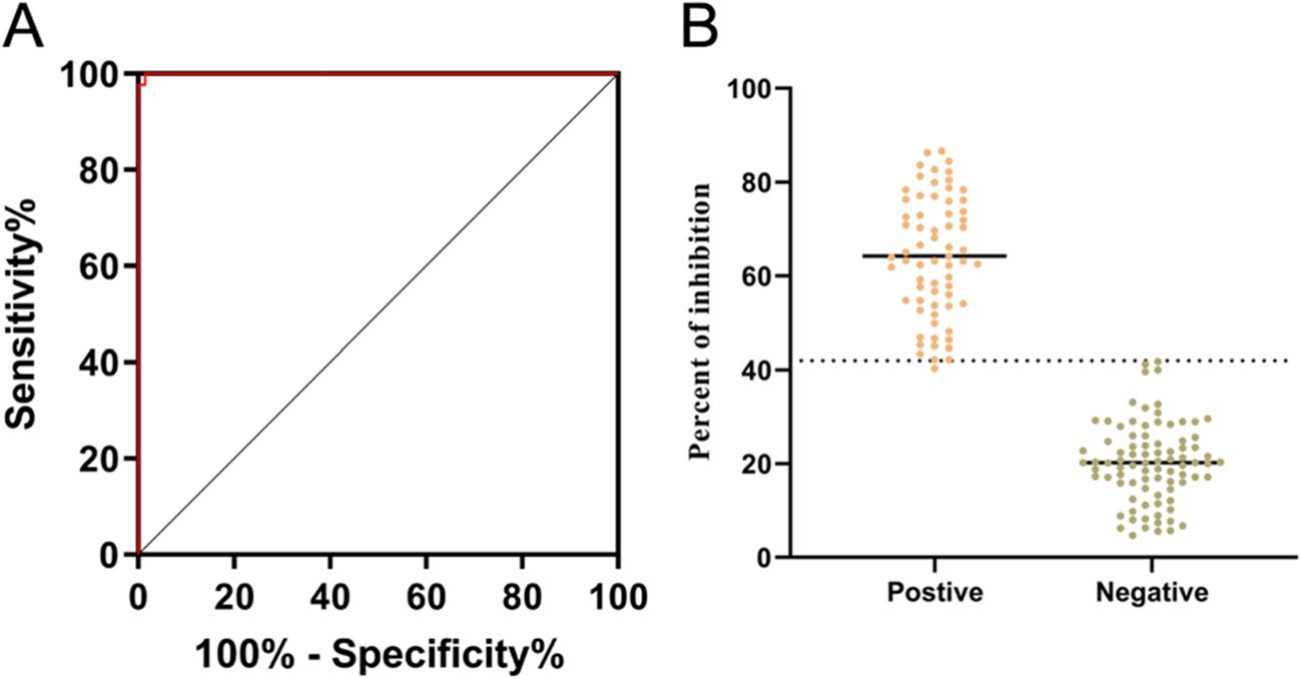
ASFV p72-based blocking ELISA analysis of serum samples. The analysis was performed on known ASFV-negative samples (*n* = 86) and known ASFV-positive samples (*n* = 67). **A** The test’s area under the curve (AUC) was 0.9997, as determined by a ROC analysis of blocking ELISA results. **B** Display a dynamic dot plot diagram illustrating the serum sample’s blocking value with a cut-off value of 41.97

The establishment of blocking ELISA involved the use of rp72 full-length protein, ASFV-positive/negative serum, and HPR-labeled 4C4 mAb. The serum was thinned to a ratio of 1:8, while the 4C4-HRP was thinned to a ratio of 1:16000. After optimizing the blocking ELISA protocol, we have enhanced the efficiency and accuracy of the experimental procedure. The method was evaluated by testing 153 samples of pig serum, consisting of 86 negative samples and 67 positive samples. The established blocking ELISA method was used to determine all serum samples and calculate the blocking rate. Then, the ROC curve of the data was analyzed, and the optimal cut-off value was calculated according to the analysis results. The established test had an area under the curve (AUC) of 0.9997 (95% CI: 99.87 to 100%) according to the ROC analysis (Fig. 6A). With a cut-off value is 41.97%, the Jorden index is the highest, which is 98.51%. At this time, the diagnostic sensitivity is 100% (95% confidence interval is 95.72%) and the specificity is 98.51% (95% confidence interval is 92.02–99.92%). It shows that the detection has high accuracy. In addition, the dot plot distribution of the blocking rate shows the cut-off value and the distribution of the sample (Fig. 6B).

### Assessment of blocking ELISA specificity, sensitivity, and repeatability

**Fig. 7:**
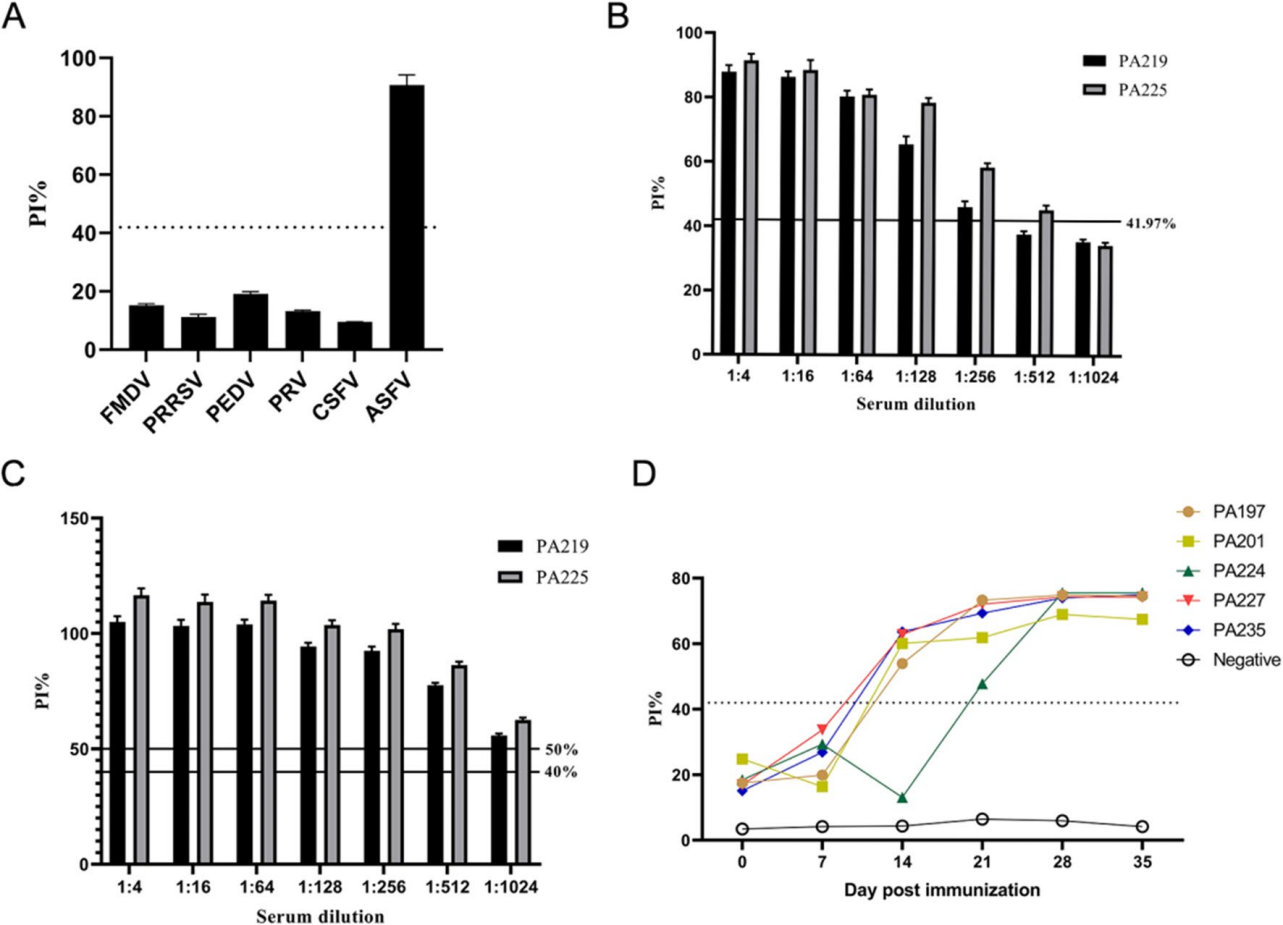
Evaluation of blocking ELISA assay. **A** Specificity analysis of the blocking ELISA. **B** The analytical sensitivity analysis of the blocking ELISA. **C** The analytical sensitivity analysis of the commercial kit. **D** Serum antibody response kinetics in pigs infected with ASFV. Serum samples were collected from six pigs infected by ASFV at 0, 7, 14, 21, 28, and 35 days post-inoculation. The dashed line indicates the threshold for blocking ELISA

According to the established blocking ELISA procedure, the positive sera of FMDV, PRRSV, PEDV, PRV, CSFV, and ASFV were detected at the same time. The results demonstrated that the established ELISA method exhibited no cross-reactivity with those sera (Fig. 7A). Two ASFV-positive sera (PA219 and PA225) were used as a sensitivity test to block ELISA, while the same assay was performed using a commercial kit. The findings indicated that the commercial kit exhibited superior analytical sensitivity at the maximum dilution of 1:1024, whereas the established blocking ELISA detection method had a detection limit of 1:256 for PA219 and 1:512 for PA225 (Fig. 7B, C). To test the repeatability, ELISA plates were blocked using both the identical antigen batch and a distinct antigen batch. The reproducibility of the results obtained by the blocking ELISA was confirmed by the consistent intra-batch variation coefficients, which ranged from 1.58 to 5.63%, and inter-batch variation coefficients, which ranged from 2.18 to 8.73%.

### Antibody response to p72 in ASFV-infected pigs

The antibody response of pigs immunized with ASFV was monitored using the established blocking ELSIA assay. The results demonstrate that seroconversion against the p72 protein was detected in the majority of pigs at 10–14 Dpi and reached its peak at 21 Dpi (Fig. 7D).

### Clinical sample test results

The sensitivity of the established blocking ELISA assay was evaluated in comparison to commercially available kits. Subsequently, clinical samples were tested using both methods to determine their coincidence rates. A total of 150 pig serum samples and 39 ASFV-infected pig serum samples were tested concurrently, resulting in an overall coincidence rate of 98.5% (Table 2). These findings demonstrate the suitability of this method for detecting ASFV clinical samples.

**Table 2:** Conformity test results for blocking ELISA

Method		Commercialization ELISA		
		Positive	Negative	Total
Blocking ELISA	Positive	38	0	38 (20.1%)
	Negative	1	150	151 (79.9%)
	Total	39 (20.6%)	150 (79.4%)	189

Po = (38 + 150)/189 = 0.995; Pe = 0.799*0.794 + 0.201*0.206 = 0.676

*K* = (0.995 − 0.676)/(1 − 0.676) = 0.985

Kappa formula for *k* = (Po − Pe)/(1 − Pe), kappa calculation results for − 1 ~ 1, but usually kappa is falling between 0 ~ 1, can be divided into five groups to indicate the consistency of the different levels: 0.0 ~ 0.20 very low consistency Slight, 0.21 ~ 0.40 fair, 0.41 ~ 0.60 moderate, 0.61 ~ 0.80 substantial, and 0.81 ~ 1 almost perfect

## Discussion

The global pig industry is facing a significant danger from ASF, as it has a mortality rate of up to 100%, a short duration of illness, and rapid transmission. Once an outbreak occurs, it typically inflicts severe damage on the local pig industry (Gaudreault et al. 2020). As there is no safe and effective vaccine and drug for the disease, the prevention and control of the disease mainly depends on accurate and effective detection technology and scientific prevention and control means (Dixon et al. 2020; Cadenas-Fernández et al. 2021). At the same time, the epitope information of viral protein is increasingly used to contribute to vaccine design and distinguish between mAbs, so as to provide a basis for the establishment and optimization of diagnostic methods (Sotelo et al. 2011; Correia et al. 2014; Fleri et al. 2017; Parvizpour et al. 2020; De-Simone et al. 2021). Therefore, the epitope localization of ASFV structural proteins also become an important link in the prevention and treatment of ASF.

Through this research, we have discovered four mAbs (5A1, 4C4, 8A9, and 5E10) that display responsiveness to rP72, thus showcasing their possible usefulness in detecting linear epitopes. Determining the antigenicity of viral structural proteins and influencing humoral immunity, epitopes play a crucial role. The identification of B-cell epitopes for ASFV is crucial for comprehending interactions between the virus and its host, as well as for the advancement of vaccines and diagnostic tools. Epitope localization was performed by truncated overlapping polypeptides. Through a series of antigenic peptide truncations, we found that ^20^ILAQDLLNSRISNIKNVNKS^39^ is recognized by both 5A1 and 4C4, which appears to be consistent with the ^31^SNIKNVNKSY^40^ epitope identified with mAbs by Yin et al. (Yin et al. 2022). Interestingly, the 5A1 and 4C4 strains showed a broader reactivity because they also recognized ^20^ILAQDLLNSR^29^ that was not recognized by reported mAbs. Meanwhile, ^263^DTQRTCTHTNPKFLSQHFPE^282^ is recognized by both 8A9 and 5E10, which is consistent with the reported 265–280 aa epitopes (Heimerman et al. 2018).

Identifying potent ASFV strains can be achieved with utmost accuracy and effectiveness through the detection of nucleic acid, which allows for the identification of both wild-type ASFV strains and gene deletion vaccine strains (King et al. 2003; Lei et al. 2020). The established dual fluorescence quantitative PCR technique, based on the 177L and p72 genes of ASFV (Lin et al. 2020), exhibits robust specificity, excellent sensitivity, and reliable repeatability. This method can effectively detect ASFV and offer a technical approach for distinguishing gene-deleted attenuated vaccines in future applications. However, in recent years, genetic regrouping of ASFV has emerged, with the emergence of low pathogenic ASFV and type I ASFV (Sun et al. 2021). The virus variants frequently result in long-lasting unusual infection in animals, exhibiting notable concealment and minimal virulence (Sun et al. 2021); this poses a challenge for the detection of nucleic acids. Thus, establishing a rapid and accurate method for detecting antibodies is crucial for effectively preventing and controlling ASF, as it combines nucleic acid detection with serum antibody detection, ensuring precision and verifiability.

The present investigation utilized the 4C4 monoclonal antibody to successfully develop a blocking ELISA technique for ASFV p72 protein; incorporating an epitope-conserved analysis, it was proved that the recognition site of this method was conservative. In laboratory settings, the blocking ELISA developed in this study using the p72 mAb showed excellent consistency and achieved optimal diagnostic sensitivity and specificity.

In summary, we generated 4 mAbs against p72 by utilizing recombinant protein expression in *Escherichia coli*. Two antigenic epitope of the prepared anti-p72 mAbs was mapped. Among them, ^20^ILAQDLLNSRISNIKNVNKS^39^, identified by 4C4 monoclonal antibody, was highly conserved in different ASFV strains. We developed a blocking ELISA based on mAb 4C4, which proved to be a promising method for rapid and convenient serum diagnosis of ASFV. The assay could function as a valuable instrument for monitoring and studying the spread of diseases in pig populations.

## Data Availability

All data generated or analyzed during this study are included in this published article.
